# Lysine-specific demethylase 2A enhances binding of various nuclear factors to CpG-rich genomic DNAs by action of its CXXC-PHD domain

**DOI:** 10.1038/s41598-019-41896-6

**Published:** 2019-04-02

**Authors:** Shiro Iuchi, Joao A. Paulo

**Affiliations:** Department of Cell Biology, Harvard Medical School, 240 Longwood Avenue, Boston, MA 20115 USA

## Abstract

The lysine-specific demethylase 2A gene (*KDM2A*) is ubiquitously expressed and its transcripts consist of several alternatively spliced forms, including *KDM2A* and the shorter form *N782* that lacks the 3′ end encoding F-box and LRR. KDM2A binds to numerous CpG-rich genomic loci and regulates various cellular activities; however, the mechanism of the pleiotropic function is unknown. Here, we identify the mechanism of KDM2A played by its CXXC-PHD domain. *KDM2A* is necessary for a rapid proliferation of post-natal keratinocytes while its 3′ end eclipses the stimulatory effect. EGFP-N782 binds to chromatin together with the XRCC5/6 complex, and the CXXC-PHD domain regulates the CpG-rich *IGFBPL1* promoter. *In vitro*, CXXC-PHD enhances binding of nuclear extract ORC3 to the CpG-rich promoter, but not to the AT-rich *DIP2B* promoter to which ORC3 binds constitutively. Furthermore, CXXC-PHD recruits 94 nuclear factors involved in replication, ribosome synthesis, and mitosis, including POLR1A to the *IGFBPL1* promoter. This recruitment is unprecedented; however, the result suggests that these nuclear factors bind to their cognate loci, as substantiated by the result that CXXC-PHD recruits POLR1A to the *rDNA* promoter. We propose that CXXC-PHD promotes permissiveness for nuclear factors to interact, but involvement of the XRCC5/6 complex in the recruitment is undetermined.

## Introduction

**Figure 1:**
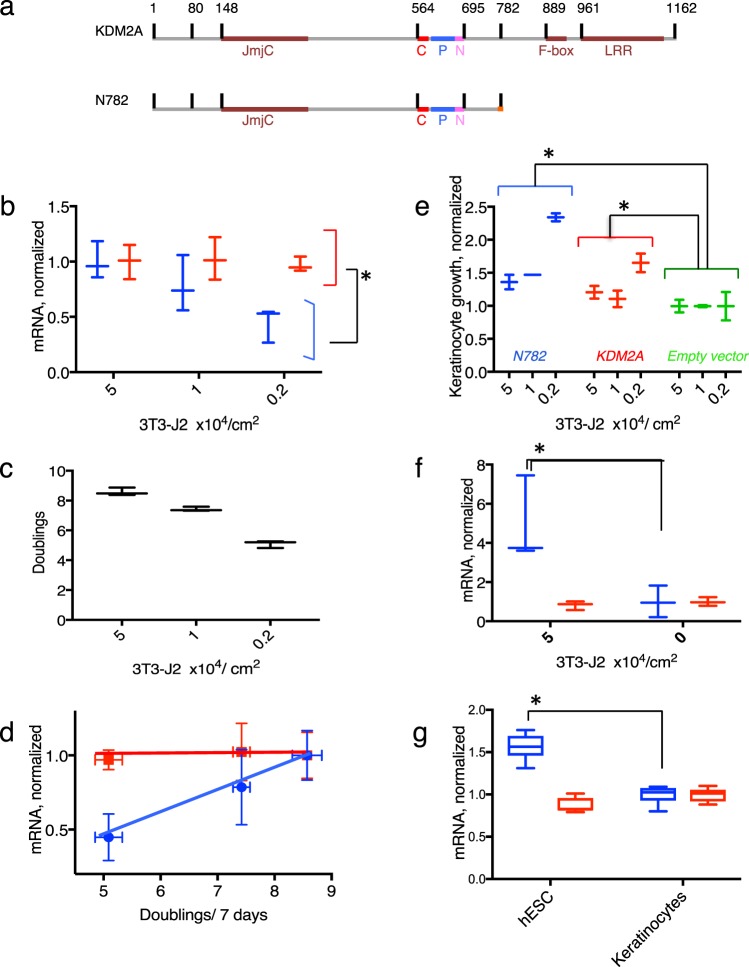
Requirement of N782 for proliferation of keratinocytes. (**a**) Illustration of KDM2A and N782. C, P, N, and LRR indicate CXXC, PHD, NLS, and leucine repeat region, respectively. (**b**) Expression of *N782* and *KDM2A* in 7-day cultivated keratinocytes at different densities of 3T3-J2. Blue and red indicate *N782* and *KDM2A*, respectively, throughout Fig. 1. p = 0.0095. (**c**) Keratinocyte proliferation under the same conditions. (**d**) Proportional relationship of *N782* to the cell proliferation rate. Mean ± SD. (**e**) Keratinocyte proliferation transduced with *N782, KDM2A*, and the empty vector (green). p = 0.0043 (*N782* vs. the control) and 0.0249 (*KDM2A* vs the control). (**f**) Expression of *N782* and *KDM2A* in the presence and absence of 3T3-J2 in EpiLife medium. p = 0.0128. (**g**) Expression of *N782* and *KDM2A* in hESC. *In Fig. 1 shows a significant difference (P < 0.05) by either ANOVA or t-test. n = 3 for (**b–f**), 2 for (**e**), and 6 for (**g**).

Dozens of protein demethylases are found in human and mammalian cells, and the demethylases are currently classified into 9 groups (KDM1 through KDM9) in the UniProt database, based on the type of both demethylase domain and other domains present in the molecule^1,2^. KDM2A is a lysine-specific demethylase that removes methyl groups from H3K36me2 and H3K36me1 coupling with cofactors Fe (II) and α-ketoglutarate^3^. It consists of JmjC, CXXC, PHD, F-box, and LRR domains (Fig. 1a). Many reports of the cellular function of KDM2A have been published since it was discovered a decade ago as the H3K36me specific demethylase. However, the fundamental role of KDM2A remains puzzling. The slow progress of investigation on KDM2A is in sharp contrast to the solid progress of that on KDM2B, which is highly similar to KDM2A and found to recruit a variant Polycomb Repressive Complex 1 (PRC1) to certain CpG Islands (CGIs) to silence the gene expression^4^.

Results of KDM2A in previous reports can be summarized in two parts: first, KDM2A binds via the CXXC domain to thousands of non-methylated CGIs that compose the majority of promoters^5–7^; second, KDM2A is involved in a variety of cellular activities such as cell division, proliferation, development, DNA repair, circadian rhythm, and cancer^8–18^. However, underlying mechanisms of KDM2A for these cellular activities are virtually unknown as the results from different groups are often conflicting. The discrepancy is especially explicit in the role on cell proliferation: some reports show that KDM2A promotes cell proliferation, while others show that it suppresses proliferation. The discrepancy appears to be derived from both multiple *KDM2A* isomers and various cells used by different groups such as normal somatic, cancer, and manipulated cells^8,12,19,20^. Cell cycle arrest at G1 phase by *KDM2A* knockdown of human apical papilla stem cells^10^ suggests that KDM2A is essential for human cell proliferation, and at the same time the results reveal that investigation of KDM2A is neither trivial nor simple.

We first encountered KDM2A during complementation experiments of poorly growing keratinocytes derived from human embryonic stem cells (hESC) H9^8^, and subsequently found that *N782*, which is a shorter isomer of *KDM2A*, consisting of the first 782 amino acid sequence (Fig. 1a), improves the poor proliferation, but the full-length *KDM2A* does not. This finding indicated that N782 has domains to stimulate cell proliferation and KDM2A has additional domains antagonizing N782 function. Current research demonstrates that N782 is necessary for rapid cell proliferation and the CXXC-PHD domain enhances recruitment of nuclear factors necessary for proliferation. We begin the investigation with characterizing *N782* as a transcript necessary for rapid proliferation of post-natal keratinocytes.

## Results

### N782 is required for rapid proliferation of post-natal keratinocytes

The *N782* mRNA transduced with the aid of a retrovirus vector improved the poor proliferation of hESC-derived keratinocytes but the full length *KDM2A* mRNA did not^8^. This result demonstrated that *N782* stimulates keratinocyte proliferation but did not prove that the transcript is required for the proliferation. If *N782* were necessary for proliferation of keratinocytes, low expression of the transcript would cause slow proliferation of cells. Therefore, we attempted to find culture conditions to lower *N782* in post-natal keratinocytes (YF29). Subsequently, we found that lower densities of lethally irradiated 3T3-J2 feeders compared to the standard density decrease *N782*. As shown in Fig. 1b, the *N782* was reduced by 60% at a density of 0.2 × 10^4^/cm^2^ of 3T3-J2 than 5 × 10^4^/cm^2^, whereas the total *KDM2A* was unaffected. Simultaneously, proliferation of keratinocytes was reduced (Fig. 1c); as a result, the proliferation rate was proportional to the expression of *N782* (Fig. 1d). Moreover, ectopic expression of *N782* improved the slow proliferation to a greater extent in the cultures containing lower density of 3T3-J2 (Fig. 1e). On the other hand, *KDM2A’s* impact was more modest under the culture conditions. Thus, expression of the *KDM2A* gene is necessary for rapid proliferation of keratinocytes, and *N782* plays the major role for the gene function. EpiLife medium is a feeder-free medium, but 3T3-J2 still increased *N782* in the medium (Fig. 1f), thereby providing further evidence that 3T3-J2 enhances the *N782* level. Importantly, hESC expressed *N782* no less than keratinocytes (Fig. 1g), suggesting that the expression mechanism and the role of *N782* are epigenetically transmitted to adult stem cells.

### The N782 protein binds to chromatin with the XRCC5/6 complex

**Figure 2:**
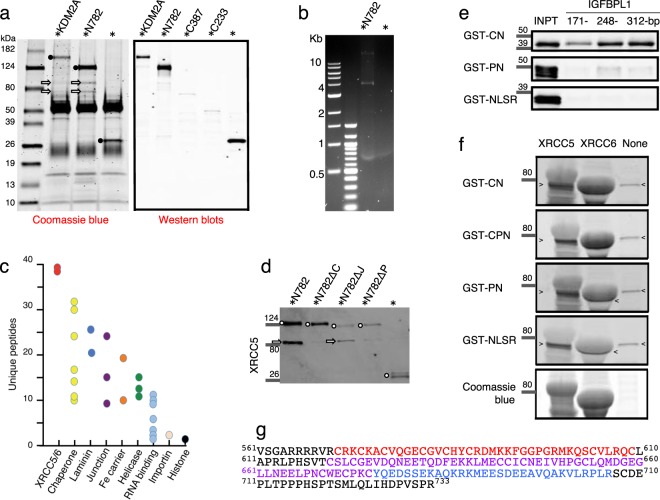
Interaction of N782 with the XRCC5/6 complex. * Indicates EGFP tag. (**a**) Left panel shows proteins immunoprecipitated with a rabbit anti-GFP antibody (black circle) and co-immunoprecipitated proteins (arrow). It contains both rabbit IgG heavy chain (50 kD wide band) and the light chain (25 kD wide band) derived from the anti-GFP antibody. Right panel shows Western blots of immunoprecipitated EGFP-KDM2A, EGFP-N782, and EGFP-KDM2A C-termini. The proteins were detected by a combination of mouse anti-GFP (primary) and goat anti-mouse IgG. (**b**) Keratinocyte genomic DNA fragments co-immunoprecipitated with EGFP-N782 and EGFP. A marker (250 or 500 ng) or sample (5 μl) was applied to each well, respectively. (**c**) Mass spectrometry of proteins included in the 70–90 kDa protein bands [proteins from top to bottom, (XRCC5 and XRCC6 in red) and (TMPO and LMNA in blue)]. (**d**) Western blotting analysis of XRCC5 (arrow) co-immunoprecipitated with N782 or the mutants (circle): Δ = deletion, C = CXXC, J = JmjC, and P = PHD. (**e**) Binding of GST-CN, GST-PN, and GST-NLSR to 3-length IGFBPL1 baits. Full-length blots are presented in Supplementary Fig. S1. (**f**) Far Western blotting analysis of the interaction of KMD2A domains with XRCC5 and 6. Probe and protein dye are on the left; cross-reaction of antibody to an *E. coli* protein, > and <. Full-length blots are presented in Supplementary Fig. S2. (**g**) Amino acid sequence of CPN domains expressed in cells (CPN, ^561^V - R^733^; CN, ^561^V- T^619^ plus ^676^Y - R^733^; PN, ^619^T - R^733^; NLSR, ^676^Y - R^733^). Typically defined domains C (red) and P (violet), and the N (blue). We have defined N by deletion analysis (unpublished).

To shed light on mechanisms involving N782, we carried out co-immunoprecipitations using an antibody to GFP and DNase-untreated cell lysates of keratinocytes transfected with pEGFP-N782. The immunoprecipitated sample was resolved by SDS-PAGE and stained with Coomassie blue (Fig. 2a left panel). It contained 2 protein bands in the range of 70–90 kDa besides EGFP-N782. The same protein bands were obtained from lysates of keratinocytes/pEGFP-KDM2A. However, the bands were weaker than those from keratinocytes/pEGFP-N782; and the band of EGFP-KDM2A itself was also weaker than that of EGFP-N782. Western blotting analysis of the similarly prepared sample also presented unbalanced-amount bands (EGFP-KDM2A < EGFP-N782) (Fig. 2a right panel). Therefore, we conjectured that lower yield of EGFP-KDM2 was derived from lower expression of the protein in keratinocytes, and turned our attention to KDM2A C-terminus that is not present in N782. Fusion of either the C-terminus (C387) with F-box and LRR or the C-terminus (C233) with LRR to EGFP strongly reduced green fluorescence in cells and also reduced immunoprecipitation yield of EGFP (Fig. 2a right panel). As F-box and LRR form the SCF (Skp1, Cullin, and F-box) ubiquitin-ligase complex^21^, the C-termini seem to have targeted either its own N-terminus (EGFP) or an unknown, but KDM2A-associated, activator. This result suggests that instability of KDM2A or lowered activators makes KDM2A ineffective in stimulating keratinocyte proliferation. Note that immunoprecipitates from keratinocytes/EGFP-N782 contained genomic DNA fragments (Fig. 2b). This means that immunoprecipitates contained proteins that interacted with N782 either directly or indirectly. Mass spectrometry of the precipitates from keratinocytes/pEGFP-N782 revealed that 70–90 kDa bands contained a pair of the X-ray repair cross-complementing protein 5/6 (XRCC5/6) complex (Fig. 2c red circle), various chaperones including HSPA5, HSPA8, and HSP90AA1, nuclear-envelope proteins LMNA (blue circle), and TMPO (blue circle) as the major proteins. The last 2 proteins indicate that N782 can bind to CGIs on boarders of lamina-associated domains (LAD)^22^. Although the immuneprecipitate also contained bands in the range of 12–14 kDa, we did not analyze them further to avoid complications resulting from small molecular mass chromatin proteins. Unlike previous reports^14,23^, neither E2F1 nor SUZ12 was found in the immunoprecipitate.

As the XRCC5/6 complex is recruited together with several other proteins to the pS2 promoter of breast cancer MCF-7 cells treated with 17β-estadiol^24^, co-recruitment of the XRCC5/6 complex does not seem to be unique to our result and rather the complex seems to be important for various regulatory elements. Accordingly, the N782-XRCC5/6 interaction was investigated further by deletion of the domains. Expression of mutant proteins was consistently lower than that of EGFP-N782 and therefore the yield of their immunoprecipitation was also lower, especially lower in JmjC deletion mutant proteins. Nonetheless, EGFP-N782ΔJmjC exhibited molecular binding activity for XRCC5 (XRCC5/EGFP-N782ΔJmjC) at a similar level as the parent protein, whereas EGFP-N782ΔCXXC completely lost the binding activity (Fig. 2d). Thus, CXXC is responsible for the interaction with the XRCC5/6 complex. Taking into account that CXXC primarily binds to CpG-rich DNA (Fig. 2e), the results suggest that N782 indirectly binds to the XRCC5/6 complex via the CXXC-CpG interaction. However, the possibility that N782 directly interacts with the XRCC5/6 complex on the chromatin still remains.

To investigate the direct interaction, size-exclusion chromatography of proteins was carried out with the purified XRCC5/6 complex alone, purified GST-CXXC-PHD-NLSR (GST-CPN) alone (NLSR, nuclear localization signal region), and a mixture of both. The result was inconclusive owing to unusual exclusion of the XRCC5/6 proteins (N. Sekiyama, personal communication). Accordingly, we took a Far Western blotting approach for the investigation by using a high concentration of XRCC5 and XRCC6 immobilized on a nitrocellulose membrane (Fig. 2f; see Fig. 2g for domains of N782). GST-domain fusion proteins bound to XRCC6 as long as the proteins contained the CXXC domain. On the other hand, both GST-CXXC-NLSR (GST-CN) and GST-PHD-NLSR (GST-PN) weakly bound to XRCC5, and GST-CPN further weakly bound to it as if CP tertiary structure inhibited the binding. These results suggest that CP can interact with the XRCC5/6 complex by alternative ways, formation of a heterotrimer with the XRCC5/6 complex via association with XRCC6 and expulsion of the complex via repelling of XRCC5.

### CP regulates gene expression at a CpG-rich promoter but not at a CpG-poor promoter

**Figure 3:**
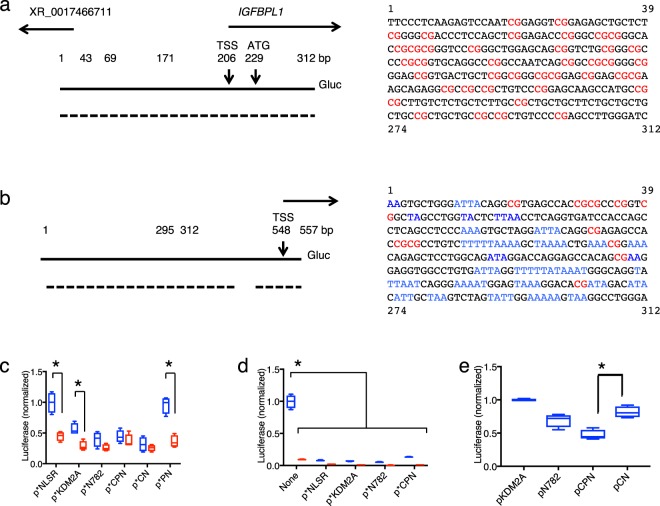
Promoters of the *IGFBPL1* and the *DIP2B* genes and their characterization. Promoters of both *IGFBPL1* (**a**) and *DIP2B* (**b**) are underlined with broken line for DNase I hypersensitive site according to ENCODE data of UCSC browser. The 5′ 67-bp fragment of the *DIP2B* consisting of a few CpGs that were bound by CXXC during the affinity purification of genomic fragments. CpG, red); and A, T, and AT stretches, blue. (**c**) Luciferase activity expressed from pIGFBPL1-312-GLuc (blue) and the empty vector pGLuc Basic 2 (red) in HEK293T cells that were co-transfected with effector plasmids encoding N782 and the domains. The activity of the culture co-transfected with pIGFBPL1-312-GLuc and pEGFP-NLSR is defined as 1.0. Small * shows EGFP. (**d**) Luciferase activity expressed from pDIP2B-557-Gluc and the empty vector. (**e**) The same-type experiment as in (**c**) but co-transfected effector plasmids were free of the EGFP tag sequence. Large * shows a significant difference by t-test (p < 0.05, n = 4).

To pursue the role of N782 and the CP domain at genomic CpG loci, we unbiasedly isolated pieces of genomic DNA by binding activity of immobilized KDM2A CXXC domain, and subsequently isolated the 312-bp promoter of the insulin-like growth factor-binding protein-like 1 (*IGFBPL1)* gene as a paradigm of CpG sequence. Selection criteria for the sequence were: (1) it is a highly CpG-rich sequence; (2) it is a short promoter sequence allowing us to easily monitor binding of the CP domain and its outcome; and (3) it is bound by CP both *in vivo* and *in vitro*. The promoter of the *IGFBPL1* gene best fitted the criteria (Fig. 3a) among the sequences that we obtained by the enrichment. Therefore, we cloned the promoter region (312 bp) from the IGFBPL1 gene, and fused it to the reporter plasmid pGluc Basic 2 (Fig. 3a). The resulting construct, pIGFBPL1-312-Gluc, was transfected into HEK293T cells (instead of inefficiently transfectable keratinocytes) and the luciferase activity was assessed. The construct induced 2.5–4 fold more luciferase activity than the empty vector, confirming that the 312-bp DNA includes the promoter although the expression was not especially high. This low expression may be associated with the character of CGIs genes proximal to LAD^22^ or with competition of various nuclear proteins for the promoter that we describe later. By using pIGFBPL1–312-Gluc, we then investigated which domains interact with the 312 bp sequence. As shown in Fig. 3c, co-transfected pEGFP-NLSR, which additionally codes the KDM2A/N-782 N-terminal 80 amino acid sequence as a linker between EGFP and NLSR, slightly reduced the luciferase activity. However, pEGFP-KDM2A substantially reduced it and furthermore pEGFP-N782 did so even more strongly. Subsequently, we found that pEGFP-CPN could severely repress the CpG-rich promoter. Although pEGFP-CN also strongly repressed it, the repression was alleviated by removal of the N-terminal EGFP (Fig. 3e). Thus, CP binds and regulates the *IGFBPL1* promoter. pEGFP-PN alone had no effect on the expression as expected from the result that PN did not bind to the *GFBPL1* promoter (Figs 2e, 3c).

To compare behavior of CP at the CpG-rich promoter with that at a CpG-poor promoter, we selected the disco interacting protein2 homolog B (*DIP2B*) gene from the collection made by affinity chromatography (see Fig. 3b legend) and fused the 557-bp promoter to the Gluc reporter gene (Fig. 3b). The first 312-bp region of the promoter contained only 11 CpG and had many A, T and AT stretches. The resulting construct, pDIP2B-Gluc-557, gave rise to 10-fold higher luciferase activity than the empty vector (Fig. 3d). The activity was strongly decreased by pEGFP-NLSR presumably via involvement of KDM2A/N782 N-terminal 80 sequence, but the residual activity was not reduced further by pEGFP-CPN. Thus, the CP domain does not have a role in regulation of the CpG-poor *DIP2B* promoter, but it has separate regulation.

### CP stimulates binding of ORC3 to a specific site of the *IGFBPL1* promoter

**Figure 4:**
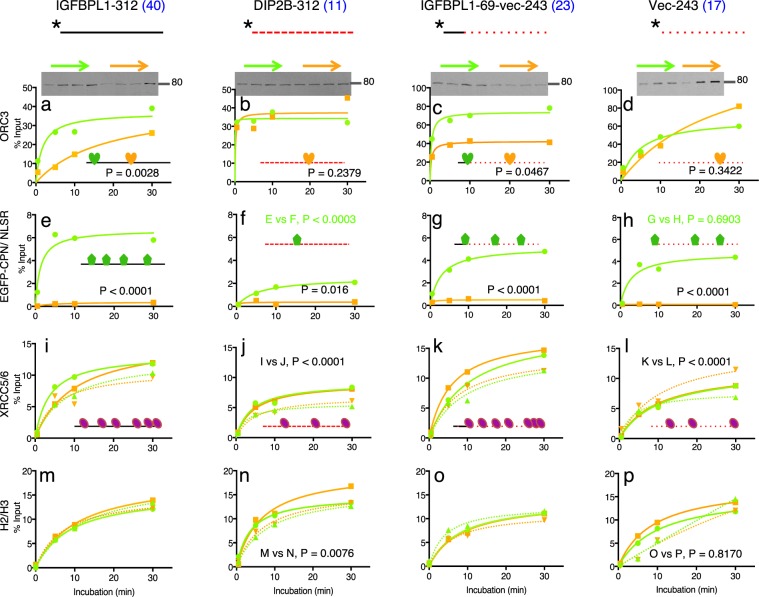
Binding kinetics of nuclear proteins to 4 different DNA baits in the presence and absence of EGFP-CPN. Investigated proteins: ORC3 in (**a**–**d**); EGFP-CPN and EGFP-NLSR in (**e**–**h**); XRCC5 (solid) and XRCC6 (dotted) in (**i**–**l**); H2 (dotted) and H3 (solid) in (**m**–**p**). Baits used for the binding are shown above the kinetics graphs along with the number of CpG (blue) counted from 5′ to 3′. *Indicates biotinylated 5′ end of baits. Green and orange kinetics indicate data obtained with EGFP-CPN-containing and EGFP-NLSR-containing nuclear extracts, respectively. Each point on the graphs represents an average of 2 independent kinetics data, but p-values of ANOVA test are calculated with all the independent points (not from the averages). Inserted cartoon suggests protein binding to the bait. Full-length blots for Fig. 4. (a through l) are presented in Supplementary Fig. S3.

CpG-rich promoters provide not only gene-specific transcription factors, but also Origin Recognition Complex (ORC) with binding sites^25,26^. In fact, ORC binds to as many as 13,500 human CpG-rich promoters^27^. On the other hand, KDM2A binds to roughly equivalent number of CGIs around promoters in mouse cells^5^. Therefore, the *IGFBPL1* promoter gave us an excellent opportunity to investigate pleiotropic regulatory feature of KDM2A with ORC. To pursue this objective, we prepared nuclear extracts from HEK293T transfected with either pEGFP-CPN or the control plasmid pEGFP-NLSR, and mixed them with immobilized IGFBPL1-312 bait, respectively, and then quantified bound ORC3 by Western blotting. ORC3 bound to the bait at a slow time-dependent rate in the control extracts (Fig. 4a). However, ORC3 bound to the bait faster in the EGFP-CPN-containing extracts. In contrast, ORC3 very rapidly bound to AT-rich promoter of the *DIP2B* (Fig. 4b) and the binding was not enhanced by CP. Interestingly, EGFP-CPN also enhanced binding of ORC3 to a hybrid 312-bp DNA containing 5′ 69-bp of the *IGFBPL1* bait (Fig. 4c), but not to the bait without the 69-bp sequence (Fig. 4d). Thus, the enhancement element was present in the 69-bp DNA of the IGFBPL1 or a sequence slightly longer than the 69-bp segment. Binding of EGFP-CPN to the baits (Fig. 4e and 4g) suggests that direct interaction of CP with the element enabled ORC3 to more efficiently bind.

Our earlier result hinted that the XRCC5/6 complex has an affinity for CpG-rich sequences (Fig. 2b,c). The notion was indeed demonstrated by the kinetics result that the XRCC5/6 complex bound to IGFBPL1-312 bait twice as much as to DIP2B-312 bait (Fig. 4i,j). Note that the XRCC5/6 complex is well established to bind to double-stranded DNA broken ends, regardless of the nucleotide composition (see inserted illustrations)^28,29^. Both H2 and H3 more slowly bound to the IGFBPL1-312 in the presence of EGFP-CPN than ORC3 did, suggesting that binding of histones does not interfere with binding of ORC3 to the *IGFBPL1* promoter (Fig. 4a,m).

### CP recruits various protein complexes essential for cell proliferation to CpG-rich promoters

**Figure 5:**
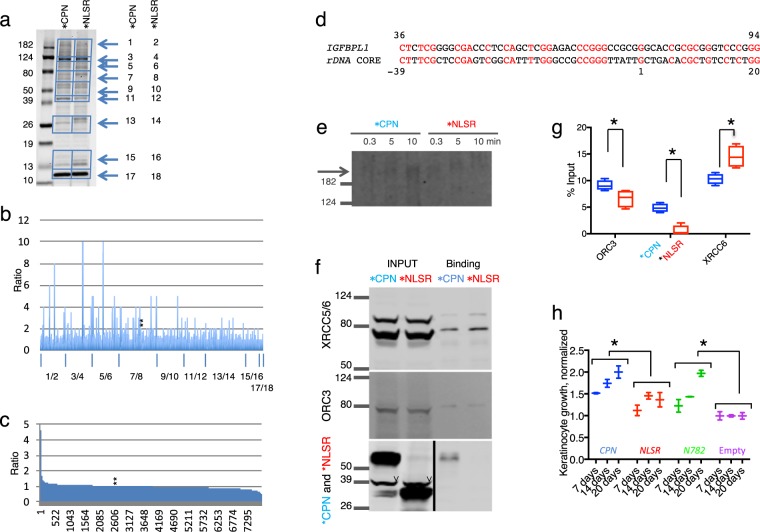
EGFP-CPN enhances nuclear protein binding to the IGFBPL1-171 bait. (**a**) SDS-PAGE analysis of proteins that bound to the bait with EGFP-CPN-containing (*CPN) and EGFP-NLSR-containing (*NLSR) nuclear extracts. Slice identification number is shown on the right. (**b**) Mass spectrometry analysis of the bound proteins. Y-axis is ratio of bound-protein between the presence and absence of EGFP-CPN, and X-axis is the slice number. Two stars (**) show the ratio for ORC3 (2.0). **(c**) Mass spectrometry analysis of the EGFP-CPN-containing and EGFP-NLSR-containing nuclear extracts. Y-axis is the same as in (**b**), and X-axis is the protein identification number. Two stars (**) show the ratio for ORC3 (1.03). (**d**) Alignment of promoters of the *IGFBPL1* and the *rRNA* genes. Nucleotides are numbered from the 5′ end of our clone for the *IGFBPL*, while TSS is shown by 1 in the *rRNA* promoter. (**e**) Time course of POLR1A binding (arrowed) to rDNA-260 promoter bait in the presence and absence of EGFP-CPN. 800 channel intensity, 6.5, was used to scan the image. Full-length blots are presented in Supplementary Fig. S4. (**f**) Binding of XRCC5/6, ORC3, and EGFP-CPN to the rDNA bait with the same extracts. (**g**) Quantification data of (**f**). 700 and 800 channel intensities were set to 4.5 and 6.5 for the scanning, respectively. * Shows a significant difference by t-test. p = 0.04 for ORC3, 0.01 for XRCC6, and <0.001 for *CPN vs *NLSR, n = 4. (**h**) Roles of CP on proliferation of keratinocytes. YF29 cultures were transduced with *CPN* (blue), *NLSR* (red), *N782* (green), and the empty vector (violet) and cultivated for up to 20 days on 0.2 × 10^4^/cm^2^ 3T3-J2 (duplicates). * Shows a significant difference by ANOVA test (p = 0.0030 for *CPN* vs *NLSR*, and 0.0002 for *N78*2 vs the control. Cell density of each measuring point was presented by fold over that of cultures transduced with the empty vector.

During the investigation of ORC3 binding, we found that the protein-band profile of the IGFBPL1-312-interacting proteins in the presence of EGFP-CPN differed from that in the control extract on SDS-PAGE gel, and the altered profile similarly occurred with the shorter bait, IGFBPL1-171 (Fig. 5a). This shorter bait does not include the region corresponding to the POLRII initiation complex binding site (Fig. 3a)^30^. Accordingly, by using it, we could investigate the recruited protein species without involvement of the major transcription complex. Following analysis of the proteins by mass spectrometry and manual data curation from the protein group, we described the recruitment of each protein by the ratio of protein amounts bound in the presence and absence of EGFP-CPN to dissociate recruited proteins from the rest. The majority of ratios for all analyzed proteins (1017 proteins) was closed to or smaller than 1, suggesting that binding of most proteins was not enhanced or excluded by CP (Fig. 5b). However, a considerable number of proteins had this ratio greater than 1. We took a ratio of 2 as indication of binding enhancement as 1.8 was the ratio for ORC3 in the same reaction period (Fig. 4a), as such, we obtained the statistic values 0.92 (mean) ± 0.33 (SD) for a group of the proteins with less than ratio 2. With this value, we subsequently calculated that 127 of 1017 proteins have ratios greater than 1.58 (0.92 + 0.33 × 2) and 94 proteins have ratios greater than 2. Thus, at least 94 proteins bound more abundantly to the CpG-rich DNA by CP. The ratio of ORC2 was slightly lower than expected in this experiment, but the mean of 2 independent experiments was 1.75 and that for the ORC-stabilizing protein LRWD^31^ was 2.1. Thus, the result was consistent even if the enhancement was not substantial. The ratio of ORC4 and ORC5 was 2 and 1.3, respectively. CDC45, required for initiation of replication and interacting with ssDNA^32^, was 4-fold more highly bound to the bait by CP. However, ORC1 was not identified even in the nuclear extracts (Fig. 5c). It seems that the cultures did not sufficiently contain G1 phase cells that are known to express ORC1^33^. MCM2-7 was virtually not recovered from the nuclear extracts (the ratio of MCM2, 3, 4, 5, 6, and 7 was 0, 0.75, 0, 0, 0, 0.41, respectively) even though all the MCM were present in the nuclear extracts. Likewise, GINS, CDC6, and CDT1 did not bind although these proteins were also present in the nuclear extracts. Taken together, our results show that ORC and the relevant proteins bind to cis elements by the action of CP during S phase without ORC1. The CP domain may replace MCM2-7 to build up the replication initiation complex and prevent second initiation in S phase^34^.

Proteins most strongly recruited by CP included POLR1A, POLR1B (Table 1), and the activator proteins (TAF1A, TAF1B, and TAF1C), as well as nucleolus proteins involved in processing of the transcripts and ribosome synthesis^35^. In addition, GNL3L required for ribosome synthesis^36^ was also recruited. The TOP3B/TDRD3 complex, which processes pre-ribosomal RNA and also activates gene expression and protects against DNA damage by breaking down the R-loop^37^, was also recruited. Notably, both UBTF (ratio, 1.0) that interacts with POLR1 via TAF1B and NUCL (ratio, 0.65) that is required for the pre-RNA processing were not recruited by CP, suggesting that the recruited proteins of this group have either a strong affinity for POLR1A/POLR1B or a direct affinity for the IGFBPL1-171 bait by other means.

**Table 1:** Proteins whose binding to the IGFBPL1-171 bait was enhanced by EGFP-CPN.

Transcription of the rRNA, the RNA processing, and ribosome synthesis
POLR1A (8), POLR1B (10), CD3EAP (2.7), TAF1A (5), TAF1B (2), TAF1C (3.5), TOP3B (2.8), TDRD3 (5), UTP15 (3), NOL11 (2), NOP2 (3), GNL3L (3), FTSJ3 (3.5), WDR36 (3), PWP2 (2.3), RPS8 (3), NOP2 (3), AGO2 (2)
Chromosome maintenance, congression, segregation, and mitosis
KIF18A (9), KIF23 (10), KIF2C (3), KIFC1 (2.7), MARK2 (4), PCID2 (3), CLASP1 (4), TPX2 (>5), KNSTRN (2), CENPQ (2), MBD3 (3), CEP170 (2), CHD3 (2.3), ARPC2 (2.5)
Replication, cell cycle, and cell proliferation marker
ORC3 (2), ORC4 (2), LRWD1 (1.8), CDC45 (4), BBX (5), MKI67 (3)
Gene silencing, gene activating, or transcription elongation
MBD3 (3), ZNF629 (>4), ZNF668 (>5), TCEB3 (4)
DNA repair
DDB2 (3), EPC2 (>4)
RNA binding
RBM27 (>4), WDR3 (2.3), ZC3H14 (4)
Cancer cell invasion and metastasis
DBN1 (3), PKN3 (>10), MPRIP (6)
Miscellaneous
KIAA0020 (3), NUFIP2 (4), TTC13 (3), TTF2 (3.5), XDH (>4), VDAC2 (2.5)

The number in parenthesis indicates enhancement factor (amount of protein bound with EGFP-CPN-containing nuclear extract/amount of the protein bound with EGFP-NLSR-containing nuclear extract). The mark > is used when protein of the control nuclear extracts did not bind to the bait at all. IGFBPL1-171 and each nuclear extract were incubated for 10 min at room temperature.

Proteins engaging in chromosome maintenance, congression, segregation, and progression of cell cycle were also highly recruited by CP. Both KIF18A and KIF2C work together to achieve the proper attachment of microtubles to the kinetochore, congression of chromosome on the spindle equator, and segregation of chromosome by depolymerizing microtubules and suppressing the process^38–41^. CLASP1 is a member of outer kinetochore proteins and important in the proper chromosome segregation processes along with TPX2 and KNSTRN^42–44^. Proteins in this class recruited by CP include CEP170, KIF18A, KIF23, KIF2C, KIFC1, and MARK2. The recruitment of all these factors explains the mechanisms how abnormal congression and segregation of chromosomes happened by mutations or knockdowns of KDM2A^15^.

Unexpectedly, CP reduced binding of the XRCC5/6 complex by half (XRCC5 was reduced to 0.52 and XRCC6 to 0.5). This result was reproduced with IGFBPL1-171 bait, but this result was not consistent with that of the IGFBPL1-312 bait (Fig. 4i). The difference between the two results can be reconciled by the observation that CP can either bind or expel the XRCC5/6 complex (Fig. 2f).

To ensure that CP enhanced the protein-DNA interaction but not nuclear protein concentration, we analyzed proteins present in the both nuclear extracts by tandem mass tag based mass spectrometry^45^. We confirmed that the recruited proteins, for example, in Table 1, were evenly present between the EGFP-CPN- and EGFP-NLSR-containing extracts and less in the former extracts (Fig. 5c). Exceptionally, however, a very small fraction of a total of 7811 proteins contained proteins that were more abundant in the EGFP-CNP-containing extracts. The fraction included STMN1 (Stathmin) and STMN2 (Stathmin 2) as well as MYCBP. Stathmin, known as a tubulin depolymerization protein, is required for proliferation and is co-related to cancer cells and the invasion^46^. This protein was 2.8-fold increased in the presence of EGFP-CPN.

An obvious skepticism on this recruitment would be why the IGFBPL1-171 was capable of binding various nuclear proteins by the CP enhancement despite this sequence being known to interact with a small number of specific proteins. We ascribe this capability to the nature of CGIs. As the islands literally have frequent repeats of CpG and high GC content in the sequences, a given CGI is highly similar to other CGIs. For example, IGFBPL1-171 was found to be 49% identical to the core element of the *rRNA* promoter without gap (Fig. 5d) to which KDM2A and the CXXC domain bind^19,20^. To prove our argument, we performed binding assay of POLR1A with rDNA-260 bait followed by Western blotting. As shown in Fig. 5e, POLR1A time-dependently bound to rDNA-260 bait in the presence of CP, whereas the binding never took place without it. We also performed binding assays of ORC3 using the same bait. ORC3 bound less to rDNA-260 bait in 10 min than to IGFBPL1-312 in terms of % input, but CP still significantly enhanced ORC3 binding (Fig. 5f,g). This result firmly supports the suggestion that CP recruits various nuclear factors to the cognate binding sites. Perhaps, different nuclear factors bound to distinct CpG sites within IGFBPL1-171 as the bait has many CpGs.

The concern of nuclear factor recruitment to IGFBPL1-171 was further addressed at cellular level. If CP enhanced only the crosstalk between genome and nuclear proteins in cells, the domain would disturb natural partnerships and result in further deterioration of the *N782*-deficient cell proliferation. Therefore, we approached such an investigation by transducing YF29 with *CPN* and the control transcripts and by allowing the cells to grow at a low density of 3T3-J2 (0.2 × 10^4^/cm). As we expected, the control keratinocytes proliferated slowly, but the cells transduced with *CPN* improved proliferation (Fig. 5h). Thus, we conclude that CP recruits the various important proteins to their natural partner CpG-rich sites in cells. The effect on cell proliferation by *CPN* was to a lesser extent than that of *N782*, suggesting that other domains of N782, including JmjC, play a supporting role for the recruitment.

Although CP enhances the binding of a variety of nuclear proteins, ectopic expression of *N782* did not fully recover cell proliferation at low densities of 3T3-J2. This suggests that keratinocytes has another pathway under control of 3T3-J2 and independent of *N782*. Factors would operate out of nucleus so that those cannot be recruited to genomic sites. Integrins are a good candidate for the factors, as they are a group of transmenbrane proteins but nonetheless stimulate proliferation of keratinocytes and inhibit terminal differentiation of the cells by direction of 3T3-J2^47,48^.

## Discussion

Here, we demonstrate that the CP domain of KDM2A enhances binding of 94 nuclear factors including ORC3 to the *IGFBPL1* promoter and further that it enhances binding of POLR1A and ORC3 to the *rDNA* promoter. This result strongly suggests that CP makes loci permissive for cognate nuclear factors to approach and bind to the loci. In other words, it is almost impossible for CP to fetch numerous nuclear factors from nucleosol to the cognate sites. Although 94 is an unusually large number, we think that this number is far below the actual number, and predict that CP enables as many nuclear proteins as the number of its binding sites. Thus, KDM2A should be able to regulate a wide variety of cellular activities even those nuclear factors that are unrelated to cell proliferation, such as circadian rhythm^18^. The CP domain seizes the basic feature of KDM2A by its broad binding to CpG-rich loci^5^ and by the permissive effect on the sites for various cognate nuclear factors.

We have not dissected the mechanisms of how CP makes the binding loci permissive, but we speculate that the domain bends or twists CpG-rich DNA and subsequently makes the loci permissive. Two scenarios can be conjectured for this process: (1) only CP is required, (2) besides CP, the XRCC5/6 complex is required. If the first were the case, then CP would eliminate adjacently sitting XRCC5/6 and promote binding of other nuclear proteins. However, if the second were the case, CP would make the sites more permissive with the XRCC5/6 complex especially with the XRCC6 subunit than CP alone. However, the XRCC5/6 complex may be expelled in the end of the reaction. Association of the XRCC5/6 complex with CGIs promoters has been recognized since the 1990’s^49^, but the role of XRCC5/6 at CpG-rich sites remains undetermined.

KDM2A was originally found to remove methyl groups from H3K36me2 and to a lesser extent from H3K36me1 by the JmjC domain^3^. However, the cellular role of this domain has not been elucidated. One report shows that the enzyme activity is involved in normal chromosome segregation^16^. The wild-type KDM2A, but not 2 mutants, the latter of which does not recognize H3K36me2 and thereby does not demethylate it in cells, recovers normal chromosome segregation in KDM2A knockdown cells. This result is consistent with our result that the CP domain of N782 recruits a number of proteins involved in chromosome segregation (Table 1) and that N782, which contains both JmjC and CP domains, more strongly enhances cell proliferation than the CP domain alone (Fig. 5h). It appears therefore that JmjC coordinates with CXXC-PHD domains to stimulate DNA-protein interactions for cell proliferation. Recently, KDM2A was found to bind HP1 through its ^779^LTVTL^803^ motif ^50^. Consequently, KDM2A enables HP1 to bind K3H9me3, and vice versa. The authors suggest that by this mechanism, KDM2A and HP1 are able to establish heterochromatin and expand the heterochromatin loci so as to silence gene expression. This pathway does not require the JmjC domain. However, repression of the *rRNA* gene by KDM2A, which occurs under starvation conditions, requires JmjC demethylation activity in addition to CXXC binding to the promoter^7,19^. The shift of KDM2A from activator to repressor states by starvation seems to be caused by modification of KDM2A via AMPK^51^. The role of KDM2A demetylase remains unresolved.

In this report, we show that KDM2As enhance the proliferation of human post-natal keratinocytes and that the short isomer N782 is virtually responsible for the effect. Further, we suggest that N782 has the same role in other human cells. The CP domain of the protein has the key role to recruit at least 94 proteins with functions ranging from replication to mitosis. Expression of *N782* is regulated. In the case of keratinocytes, expression of *N782* is turned off accompanying poor proliferation. However, 3T3-J2 feeders activate the expression and allow keratinocytes to proliferate at a rapid rate. Our findings help us understand both the basic mechanisms of human cell proliferation and feeding mechanism of 3T3-J2. Feeding of 3T3-J2 is essential for medical teams to prepare autologous skin grafts for treatment of life-threatening skin afflictions^52,53^.

## Methods

### Cell culture, transfection, and transduction

Post-natal keratinocytes (YF29) and hESC (H9) were routinely cultivated on feeder cells as described^54,55^. HEK293T cells were cultivated in DMEM supplemented with 10% fetal bovine serum. Cells used in this study, the cultivation, and the handling procedures have been approved by Harvard University Committee on Microbiological Safety and Embryonic Stem Cell Research Oversight Committee. We carried out experiments under the guidelines: NIH rDNA Guidelines; and the Federal Occupational Safety and Health Administration Bloodborne Pathogen Standard. Our experiments neither involved humans nor animals as the cultures had been established by other scientists before we started this investigation. For transfection, YF29 was transferred to a dish with feeder-free EpiLife (Invitrogen) one day before transfection, and HEK293T was similarly transferred to a dish with fresh DMEM/FBS. Ten-μg plasmid/10-cm dish and 5-μg plasmid/6-cm dish) were transfected with X-tremeGENE 9 DNA transfection reagent (Roche). Transfected keratinocyte and HEK293T cultures were incubated for 19 to 20 h and for 2 days, respectively. Transfection efficacy of YF29 was as poor as 5% and highly variable from one to another, while that of HEK293T was about 30%. Longer incubation did not improve transfection efficacy of keratinocytes. For measurement of promoter activity, HEK293 cells were inoculated in wells of a 96- well plate; and after one day, the cultures were transfected with 0.1-μg pIGFBPL1-312-GLuc and 0.2-μg plasmid encoding KDM2A or its derivatives. pIGFBPL1-312-GLuc was replaced with the empty vector, pGLuc-Basic 2, as the control. Plasmid pDIP2B-557-Gluc was used for estimation of the *DIB2B* promoter activity. Supernatant of the cultures were harvested and used for the luciferase activity assay with the BioLuc Gaussia Luciferase Assay kit (NEB). Keratinocytes was transduced with virions prepared by the method^8^. However, in this study selection for puromycin was skipped to avoid puromycin-induced terminal differentiation of post-natal keratinocytes.

### Polymerase chain reaction

DNA sequences of interest were amplified using the Q5 Hot Start High-Fidelity 2x Master Mix (NEB). Primers are described in Tables 2 and 3. In general, template was initially denatured for 30 s at 98 °C and for 10 s before each cycle, annealing was performed at 0–2 °C higher temperature than the Tm of the primers, and polymerization was performed for 20 s per kbp at 72 °C. qPCR was carried out with AB-4167/A (Thermo Scientific). Ten-μl reaction mixture was initially heated for 15 s at 95 °C and 15 s for each cycle, annealing was performed for 30 s at 67 °C, and polymerization was performed for 30 s at 68 °C. Forty cycles were performed. *KRT14* and *G3PDH* mRNA were amplified for normalization of *N782* and *KDM2A* mRNA within keratinocytes and between keratinocytes and hESC, respectively.

**Table 2:** Oligonucleotides used in this work.

Primer name	Primer sequence	Strand
Olit146	ttactccaccggctgataaaccag	Forward
Olit149	gggccagctgctcctccac	Reverse
Olit169	cttgtcgcagcaccatgg	Reverse
Olit170	agtcgaattccagctgctgcctcccc	Forward
Olit171	gactctcgagttatgtgactgagtgaggcagtctg	Reverse
Olit172	cggccgctctagaactag	Forward
Olit173	gaattcctgcagcccg	Reverse
Olit174	cggactcagatctcgatggaacccgaagaagaaagg	Forward
Olit175	gtaccgtcgactcaatttttgtccaaagtctcttgt	Reverse
Olit176	tgtgatgtcaaggcctgccag	Reverse
Olit177	tgcagtcccagctcagcatgaaagca	Forward
Olit180	aaacggcggcagttgct	Forward
Olit181	gtaccgtcgacgtgtgtcttagctgatcttctgtatcag	Reverse
Olit182	ctctctgctcctcctgttcgac	Forward
Olit183	tgagcgatgtggctcggct	Reverse
Olit184	cggactcagatctcacgggaaaaggagaacaatcc	Forward
Olit185	cggactcagatctcttggacaaaaattgacttgagtaggt	Forward
Olit196	ctctccttgcacacagg	Reverse
Olit197	ttggcacccagactgc	Forward
Olit198	gtacttctgcactttagggtac	Reverse
Olit199	ttgcatagcttcaacatccc	Forward
Olit200	tgtgactgagtgaggcag	Reverse
Olit201	taccaggaggacagctc	Forward
Olit208	cactgaattcttaccaggaggacagctc	Forward
Olit209	ttaaaggatccgatgaccgagtcaccatac	Reverse
Olit250	cactgaattctgtgtcaggagccagac	Reverse
Olit253	cactgaattctacatgttccctctgtgga	Forward
Olit254	attcgagctccgtcgacaatggtgcggtcggg	Forward
Olit255	tgctcgagtgcggccgcactatatcatgtccaataaatcgtccacat	Reverse
Olit256	attcgagctccgtcgacaatgtcagggtgggagtcata	Forward
Olit257	tgctcgagtgcggccgcatcagtcctggaagtgcttg	Reverse
Olit276	attggaattccctcaagagtccaatcgga	Forward
Olit279	acataagcttgaactcgcaagggcc	Reverse
Olit280	ggtggcgaccggtag	Reverse
Olit281	atggaacccgaagaagaaagga	Forward
Olit288	5′-/5Biosg/ttccctcaagagtccaatcg	Forward
Olit289	gatcccaaggctcggg	Reverse
Olit293	tcgtcctgcagttcattca	Reverse
Olit294	agcagagacaagcgcg	Reverse
Olit297	agcagtcaccgctcc	Reverse
Olit309	5′-/5Biosg/aagtgctgggattacagg	Forward
Olit310	tcccaggccttactttttc	Reverse
Olit313	5′-/5Biosg/agcttgtatatccattttcggatc	Forward
Olit314	tacacgaagcttgtcagatccgctagcg	Forward
Olit315	ggctaaactcgaggccctctacaaatgtggtatggc	Reverse
Olit318	ttagaattctggggttgaccagaggg	Forward
Olit319	gttggatccgtcaccggtaggccaga	Reverse

**Table 3:** Usage of primers.

Primers	Template	Object or construct
Olit146/Olit176	first strand cDNA	qPCR for *KDM2A* mRNA
Olit180/Olit169	first strand cDNA	qPCR for *N782* mRNA
Olit149/Olit177	first strand cDNA	qPCR for *KRT14* mRNA
Olit182/Olit183	first strand cDNA	qPCR for *GAPHD* mRNA
Olit170/Olit171	pEGFP-N782	pET-CXXC
Olit172/Olit173	pBluescript KS(+)−	Amplification of genomic
	genomic DNA fragments	DNA fragments in the vector
Olit174/Olit181	KDM2A in pMarXG7	pEGFP-KDM2A
Olit174/Olit175	KDM2A-N782 in pMarXG7	pEGFP-N782
Olit184/Olit181	pEGFP-KDM2A	pEGFP-C387
Olit185/Olit175	pEGFP-KDM2A	pEGFP-C233
Olit196/Olit197	pEGFP-N782	pEGFP-N782 ∆CXXC
Olit198/Olit199	pEGFP-N782	pEGFP-N782 ∆JmjC
Olit200/Olit201	pGEX-CXXC-PHD-NLSR	pGEX-CN
Olit208/Olit209	pEGFP-N782	pEGFP-NLSR– > pGEX-NLSR
Olit250/Olit209	pEGFP-N782	pEGFP-CPN and pGEX-CPN
Olit250/Olit209	pEGFP-N782 ∆PHD	pGEX-CN
Olit250/Olit209	pGEX-CXXC-NLSR	pEGFP-CN
Olit253/Olit209	pEGFP-N782	pEGFP-PN and pGEX-PN
Olit254/Olit255	First strand cDNAs	pET-XRCC5
Olit256/Olit257	First strand cDNAs	pET-XRCC6
Olit276/Olit279	Keratinocyte genomic DNA	pIGFBPL1-687-Gluc–>
Olit280/281	pEGFP-CXXC-NLSR	pCN
Olit280/281	pEGFP-PHD-NLSR	pPN
Olit280/281	pEGFP-CXXC-PHD-NLSR	pCPN
Olit280/281	pEGFP-NLSR	pNLSR
Olit280/281	pEGFP-KDM2A	pKDM2A
Olit280/281	pEGFP-N782	pN782
Olit288/Olit297	pIGFBPL1-687-GLuc	IGFBPL1-171 bait
Olit288/Olit294	pIGFBPL1-687-GLuc	IGFBPL1-248 bait
Olit288/Olit289	pIGFBPL1-687-GLuc	IGFBPL1-312 bait
Olit288/Olit293	pIGFBPL1-312 ΔXmal-GLuc	hybrid-312 bait
Olit309/Olit310	pDIP2B-GLuc	DIP2B-312 bait
Olit313/Olit293	pIGFBPL1-312 ΔXmal-GLuc	Vec-243 bait
Olit314/Olit315	pCPN	pMarXG–> pMar-CPN
Olit314/Olit315	pNLSR	pMAXG–> pMar-NLSR
Olit318/Olit319	Keratinocyte genomic DNA	*rDNA* promoter

### Construction of plasmids

Coding regions of the *KDM2A* and *N782* genes were amplified by PCR and ligated to the BglII/SalI sites of pEGFP C2, so that the N-termini of the proteins were fused to the C-terminus of EGFP (pEGFP-KDM2A and pEGFP-N782). From these, additional plasmids were constructed by deletion and insertion by using restriction enzymes or PCR. DNA encoding KDM2A domains was cloned into pEGFP-N80 (a pEGFP derivative with the sequence for the first 80 amino acids of KDM2A as a linker) or pGEX-5X-3 and pET-28C(+) for production of the domains in human cells or *E. coli*. The coding regions of the *XRCC5* and *XRCC6* genes were amplified by PCR using a YF29 cDNA pool as templates that were prepared using the ProtoScript first strand cDNA synthesis kit (NEB) and cloned into pET-28C(+) at the HindIII restriction site. XRCC5 and XRCC6 expressed from the resulting plasmids in *E. coli* specifically reacted to mouse-monoclonal antibodies to XRCC5 and XRCC6, respectively.

### Cloning of genomic DNAs

We unbiasedly cloned human CpG-rich loci^56^. Briefly, Sau3AI digest fragments of a YF29 genomic DNA were collected by affinity chromatography with purified and immobilized 6x his-tagged KDM2A CXXC, and ligated to BamHI site of pBluescriptKS (+) and amplified by PCR. The product was ligated back to the empty vector. This amplification procedure was carried out only once not to amplify CpG-rich sequences methylated in cells. The prepared plasmids were transformed and amplified in *E. coli*, and the randomly isolated. Subsequently the inserts were identified by sequencing. Based on the identification, the promoter regions of the *IGFBPL1* and the *DIP2B* genes were cloned, and their validity was confirmed by sequencing. The 455-bp sequence of the *IGFBPL1* has been deposited (GenBank accession number, KX688890). The sequence of the *DIP2B* promoter was identical to that from 50504472 through 50504994 of GenBank ID NC_000012.12. The *rDNA* promoter (−179 through + 81) was cloned by PCR, whose sequence was the same as that in GenBank ID MF164270.1

### Immunoprecipitation

Keratinocyte cultures were lysed in 50 mM Tris-HCl (pH 7.4) with 1% Triton X-100, 10% glycerol, 150 mM NaCl, 1 mM EDTA, 0.2 mM sodium orthovanadate, 1× EDTA-free Complete Protease Inhibitor Cocktail (Roche), and 1 mM PMSF in a cold room. The lysates were pre-cleared for 1 h with protein A magnetic beads (NEB) that had been charged with normal-rabbit-IgG (Santa Cruz). The cleared supernatant was then mixed with protein A magnetic beads that had been charged with rabbit anti-GFP (Acris, SP3005P) and rotated for 3 h. Then, beads bearing protein complexes were collected and washed twice with the lysis buffer. The immunoprecipitated complexes were released from the beads by heating in a 3× loading buffer.

### Preparation of extracts

We prepared nuclear extracts, slightly modifying the reported procedure^57^. The nuclear fraction was suspended in nuclear extraction buffer consisting of 50 mM Tris-HCl pH 7.4, 1% Triton X-100, 10% glycerol, 150 mM NaCl, 0.2 mM sodium orthovandate, 1x EDTA- free Complete Protease Inhibitor Cocktail, 1 mM PMSF, 5 μl/ml RNase (Roche), and 25 μM ZnSO_4_. The suspension was sonicated 3 times for 3 s each time. The lysates were centrifuged after addition of EDTA to 1 mM and the supernatants were used as nuclear extracts. Cell extracts containing XRCC5, XRCC6, and GST-fusion probes were prepared from *E. coli* BL21 (DE3) or a derivative in which each protein were overexpressed. Collected bacterial cell pellets were subjected to freezing-thawing 3 times, then suspended in the nuclear extraction buffer containing 1 mg/ml lysozyme. The lysates were sonicated and the supernatants were saved. When necessary, the pellets were suspended in the same buffer and homogenized by brief sonication.

### DNA-protein interaction

Bait used in this study was prepared by PCR including a 5′ end biotinylated forward primer and a backward primer. Four pmol bait was bound to hydrophilic streptavidin magnetic beads (NEB, S1421S), and incubated with 200 µg nuclear extracts in 50 µl up to 30 min at room temperature. The beads were washed twice with 100 µl extraction buffer. The bound proteins were released in 20-µl 1.5X sample buffer by heating for 5 min at 100 °C, and collected immediately for subsequent SDS-PAGE. For mass spectrometry, the IGFBPL1-171 bait (4 pmol) was incubated with 500 µg nuclear extracts in 72 µl for 10 min. Binding of GST-CN to IGFBLL1 baits similarly performed in the presence of 10 units of poly[d(A-T)] per reaction by 10-min incubation. Protein samples interacted with DNA were separated on 4–20% Gold Precast Gels by SDS-PAGE. Then, proteins on the gel were stained with ProtoBlue Safe Colloidal Coomassie G-250 (National Diagnostics) or transferred to a nitrocellulose membrane (Bio-Rad) for Western blotting. Both stained gel and nitrocellulose membrane were scanned using a LI-COR Odyssey Imager. Resolution and Quality were set to 169 μm and medium, respectively. Seven hundred and 800 channel intensities were set to 3.5 and 5.0, respectively, unless otherwise indicated. ImageStudioLite2 was used to collect images and quantitate data. The following antibodies were used for Western blotting: rabbit anti-ORC3 (Thermofisher, PA5-28083); rabbit anti-POLR1A (Thermofisher, PA5-37156); mouse anti-XRCC5 (Thermo Scientific, MA5-15873); mouse anti-XRCC6 (Abnova, H00002547-M01), rabbit polyclonal anti-H3 (Thermofisher Scientific, PA-16183), mouse anti-H2B (Biolegend, 688702), anti-GFP monoclonal antibody (Clontech), rabbit anti-GFP (Acris, SP3005P); goat anti-rabbit IgG (H + L), IRDy 800 conjugated (Rockland, 611-132-122); and IRDye 680RD goat anti-mouse IgG (H + L) (LI-COR, 926-68170).

### Protein-protein interaction

Far Western blotting was used to investigate interaction of KDM2A domains with XRCC5 and XRCC6. Both XRCC5 and XRCC6 were prepared from *E. coli* particulate fractions and immobilized onto nitrocellulose membrane after SDS-PAGE, and then renatured by the method of Einarson^58^. The proteins on the membrane were incubated overnight at 4 °C in the HEPES buffer (without guanidine hydrochloride) with 150 mM NaCl and 5% non-fat dry milk for blocking, and then incubated for 2 h at room temperature in the HEPES buffer consisting of 150 mM NaCl/1% non-fat milk with bacterial extracts (soluble fractions) containing each probe: GST-CN, GST-PN, GST-CPN, or GST-NLSR. The membrane was washed 4 times in the HEPES buffer/NaCl. Eventually, the probes on the membrane were immunologically identified with goat-anti GST (Pharmacia).

### Mass spectrometry

For identification of DNA bait- and N782-binding proteins, mass spectrometry was performed by the previously described procedure^59^. For identification of total nuclear proteins, tandem mass tag-based mass spectrometry was performed^45^. The mass spectrometry proteomics data have been deposited to the ProteomeXchange Consortium via the PRIDE^1^ partner repository with the dataset identifier PXD012941.

### Analytical software

BLAST, Cn3D, and GenBank database at National Center for Biotechnology Information and USCS Genome Browser at University of California, Santa Cruz were used for various computational analysis of proteins and nucleic acids. The human Uniprot database was used for identification of bait-CPN-binding proteins. Lasergene (DNASTAR) was used for sequence alignment. Microsoft Excel software and GraphPad Prism were used for statistical analysis. t- and ANOVA-test: p < 0.05 indicates a significant difference.

Sequence and mass spectrometry data obtained during current study are available as stated in the Methods section.

## Supplementary information

Dataset 1
