# Supplementary material for: Lysine-specific demethylase 2A enhances binding of various nuclear factors to CpG-rich genomic DNAs by action of its CXXC-PHD domain
Source: Sci Rep. 2019 Apr 2;9:5496. doi: 10.1038/s41598-019-41896-6 (PMC6445129; doi:10.1038/s41598-019-41896-6)
Supplement: Supplementary file 1 — Dataset 1 [file 41598_2019_41896_MOESM1_ESM.pdf]

Lysine-specific demethylase 2A enhances binding of various nuclear factors to CpG-rich genomic DNAs by action of its CXXC-PHD domain

Shiro Iuchi* and Joao A. Paulo

Supplementary Figure 1 for text Fig. 2e

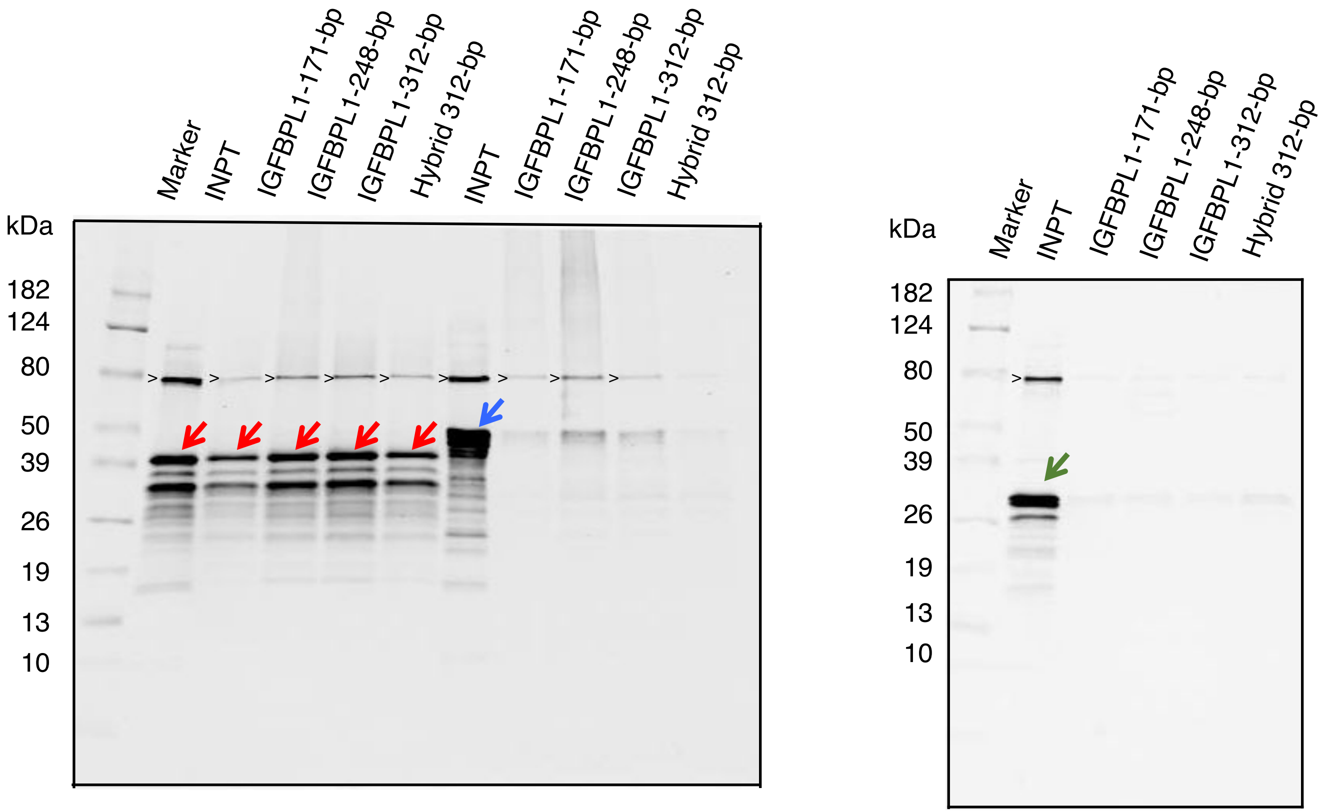

Red, GST-CN; Blue, GST-PN; and Green, GST-NLSR. Hybrid 312 = IGFBPL1-69-vec-243 (Fig. 4). Partially degraded GST-CN also binds the baits. > shows cross reaction with *E. coli* protein.

Supplementary Figure 2 for text Fig. 2f

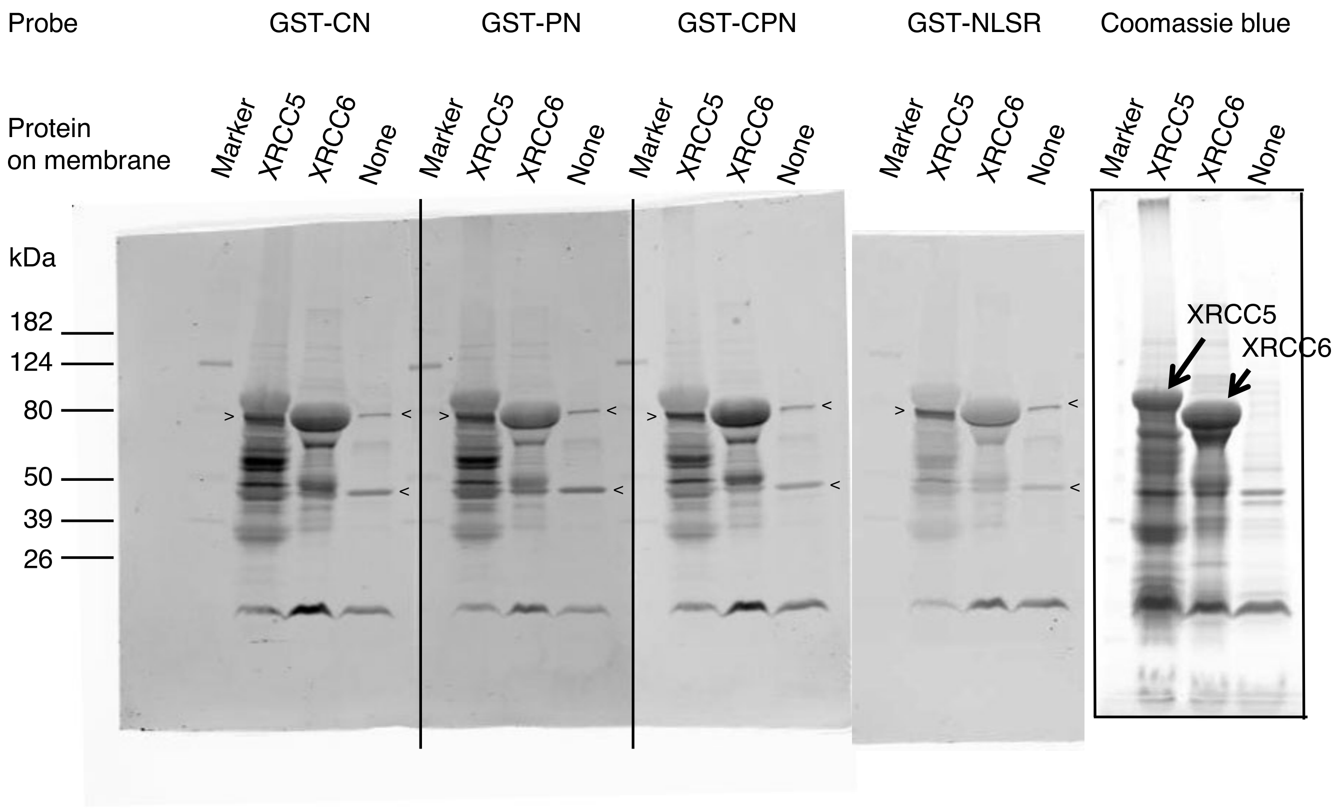

Nitrocellulose membrane with renatured proteins was cut along straight lines (black). A piece of the membrane was incubated with a probe and subsequently immunostained. Eventually, the membrane pieces were assembled and scanned for the probe. The membrane for GST-NLSR probe was prepared separately but in parallel. Intact XRCC5 and XRCC6 are indicated by an arrow

Supplementary Figure 3 for text Fig. 4a through 4l

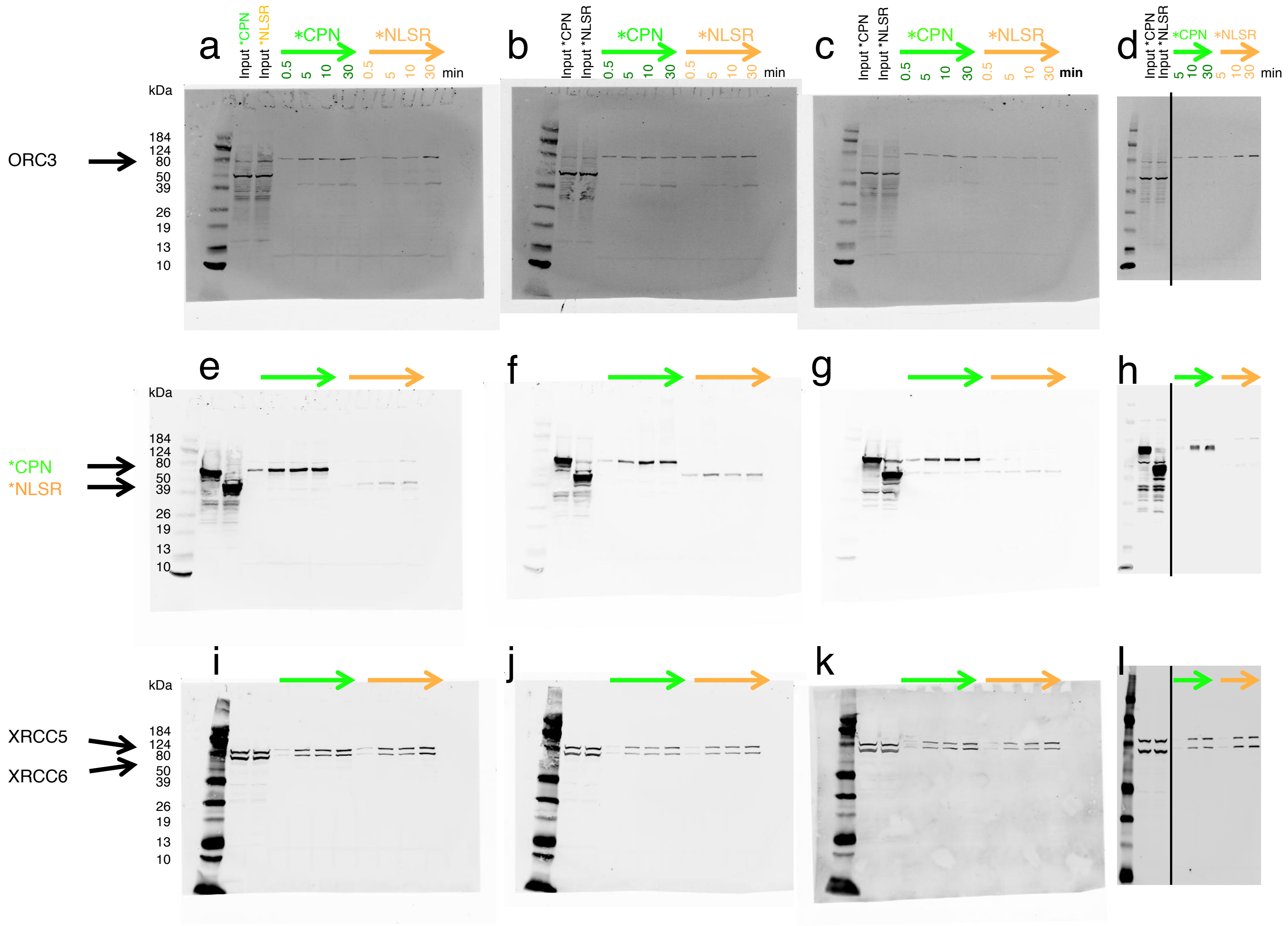

Time course of nuclear extract protein binding to 4 different baits (**a, e, i** to IGFBPL1-312; **b, f, j** to DIP2B-312; **c, g, k** to IGFBPL1-69-vec-243; and **d, h, l** to Vec-243 in the presence (green) and absence (orange) of EGFP-CPN. Star (*) indicates fusion with EGFP.

Supplementary Figure 4 for text Fig. 5e

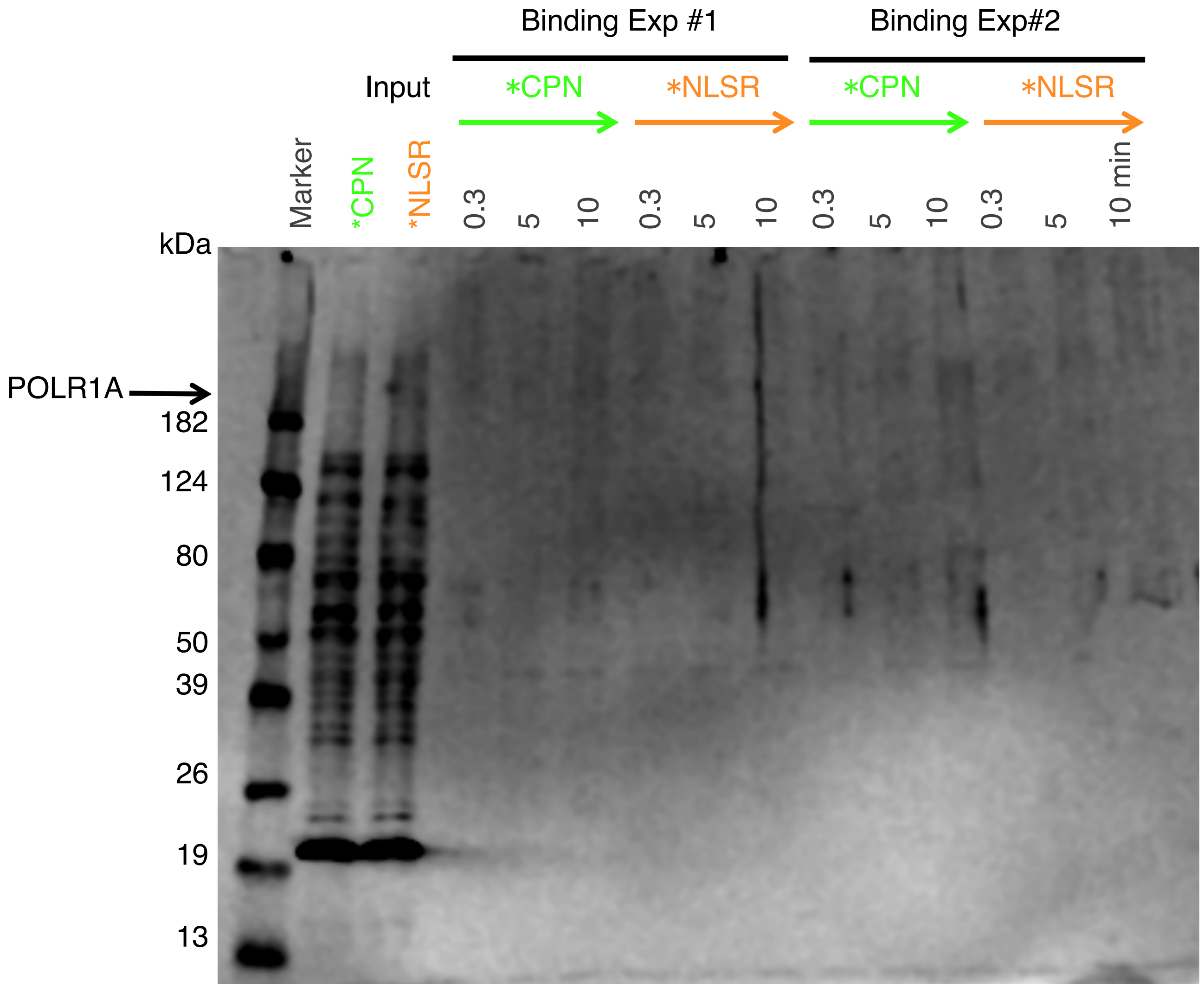

POLR1A was highly degraded in nuclear extracts, but it time dependently binds the *rDNA* promoter bait in the presence of EGFP-CPN (green). Star (*) indicates EGFP.
